# P007. Inhibition of monoacylglycerol lipase activity modulates the activation of brain structures relevant for migraine pathogenesis

**DOI:** 10.1186/1129-2377-16-S1-A165

**Published:** 2015-09-28

**Authors:** Rosaria Greco, Tiziano Bandiera, Antonina S Mangione, Chiara Demartini, Giuseppe Nappi, Giorgio Sandrini, Daniele Piomelli, Cristina Tassorelli

**Affiliations:** Laboratory of Neurophysiology of Integrative Autonomic Systems, Headache Science Centre, “C. Mondino” National Neurological Institute, Pavia, Italy; Drug Discovery and Development Department, Fondazione Istituto Italiano di Tecnologia, Genova, Italy; Department of Brain and Behavioural Sciences, University of Pavia, Pavia, Italy

## Background

Experimental evidence shows that the anti-nociceptive action of endocannabinoids, related to the modulation of the trigeminovascular system activity, may be helpful for prompting new targets for the treatment of migraine. URB602 is an inhibitor of monoacylglycerol lipase (MAGL), a key enzyme in the hydrolysis of the endocannabinoid 2-arachidonoylglycerol (2-AG). URB602 induces analgesia in animal pain models not related to migraine, but there is no pre-clinical information as regards to its potential effect in migraine pain.

## Aim

To evaluate whether URB602 administration interferes with the level of activation of brain structures involved in migraine.

## Methods

Nitroglycerin (NTG) induces neuronal activation in a specific subset of brain nuclei that are considered relevant for the development of migraine attacks. In this study we evaluated the changes caused by URB602 in NTG-induced neuronal activation. Male Sprague Dawley rats were treated with NTG (10mg/kg, i.p.) followed by URB602 (2mg/kg, i.p.) or vehicle (DMSO, 1ml/kg, i.p.). Their brains were processed for the detection of c-Fos protein, used as an indicator of brain activation.

## Results

URB602 alone did not change Fos expression in the brain nuclei under evaluation. When administered 3 hours after NTG, URB602 reduced NTG-induced Fos expression in all the cerebral areas that were examined, with a significant effect in nucleus trigeminalis caudalis and ventrolateral column of periaqueductal grey.

## Conclusions

The inhibition of MAGL activity, with the theoretical increase of central content of 2-AG, may modulate the activation of structures involved in pain perception and pain integration in an animal model specific for migraine.

